# Safety and effectiveness of everolimus in maintenance kidney transplant patients in the real-world setting: results from a 2-year post-marketing surveillance study in Japan

**DOI:** 10.1007/s10157-021-02024-9

**Published:** 2021-02-11

**Authors:** Naomi Hayase, Mariko Yamada, Shuhei Kaneko, Yoko Watanabe

**Affiliations:** 1 Novartis Pharma K.K. Medical Division, 23-1, Toranomon 1-chome, Minato-ku, Tokyo, 105-6333 Japan; 2 Patient Safety Japan Re-Examination Department, Novartis Pharma K.K., Tokyo, Japan; 3 Biostatistics Pharma, Integrated Biostatistics Japan, Clinical Development & Analytics Japan, Novartis Pharma K.K., Tokyo, Japan; 4 Immunology, Hepatology & Dermatology Clinical Development Department, Clinical Development & Analytics Japan, Novartis Pharma K.K., Tokyo, Japan

**Keywords:** Everolimus, Maintenance kidney transplant patients, Renal impairment, Observational study, Post-marketing surveillance

## Abstract

**Background:**

Data on real-world use of everolimus (EVR) in Japanese maintenance kidney transplant (KTx) patients are limited. This post-marketing surveillance study was conducted to assess the safety and effectiveness of EVR, and identify factors affecting renal impairment.

**Methods:**

Adult maintenance KTx patients were enrolled within 14 days of initiating EVR. Patient medical data were collected using electronic data capture case report forms at 6 months, 1, and 2 years after initiating EVR, or at discontinuation.

**Results:**

All patients receiving EVR in Japan during the surveillance period were enrolled (*N* = 263). Mean time from transplantation to EVR initiation was 75.7 months. Decreased renal function (31.56%) was the primary reason for initiating EVR. In combination with EVR, the mean daily dose of tacrolimus and cyclosporine could be reduced to ~ 79 and ~ 64%, by 2 years, respectively. Incidences of serious adverse events and adverse drug reactions were 15.97 and 49.43%, respectively. Two-year graft survival rate was 95.82% and low in patients with baseline estimated glomerular filtration rate (eGFR; modification of diet in renal disease) < 30 mL/min/1.73 m^2^ (69.57%; *P* < 0.0001) and urinary protein/creatinine ratio (UPCR) ≥ 0.55 g/gCr (84.21%; *P* = 0.0206). Throughout the survey, mean eGFR values were stable (> 55 mL/min/1.73 m^2^). Renal impairment was influenced by patient and donor age, eGFR, and UPCR at baseline.

**Conclusions:**

No new safety concerns for the use of EVR in adult maintenance KTx patients were identified. Early EVR initiation may be considered in these patients before renal function deterioration occurs.

**Supplementary Information:**

The online version contains supplementary material available at 10.1007/s10157-021-02024-9.

## Introduction

Current immunosuppressive protocols with calcineurin inhibitors (CNIs) provide good short-term efficacy but their long-term use is associated with chronic nephrotoxicity [1, 2], CNI arteriolopathy [3], diabetes [4, 5], and cardiovascular complications [6]. Thus, immunosuppressive strategies that can facilitate CNI minimization/elimination, while maintaining long-term anti-rejection efficacy are being developed [7–9].

Several studies have reported the efficacy and safety of everolimus (EVR) as a maintenance immunosuppressant in kidney transplant (KTx) patients [10–14]. The main reasons for switching to an EVR-based regimen were interstitial fibrosis and tubular atrophy, CNI-associated nephrotoxicity, cancer, viral infections, and generalized vascular disease [10, 11].

Although EVR has been approved in Japan for “inhibition of graft rejection in kidney transplantation” in 2011, data on clinical experience with EVR in Japanese maintenance KTx patients are limited. In a previous post-marketing surveillance (PMS) study in Japan, efficacy and safety of EVR in both de novo and maintenance KTx patients was reported up to 2 years. However, no data on renal function, influence of baseline characteristics on efficacy and safety were reported [14]. The current PMS study was planned to assess the safety and effectiveness of EVR in adult maintenance KTx patients and to identify factors responsible for subsequent renal impairment.

## Patients and methods

### Survey design and population

This was a 2-year, observational, non-interventional, multicenter, PMS study conducted between September 2014 and August 2018 by a central registration system. KTx patients aged ≥ 18 years receiving EVR in the maintenance period were enrolled from December 2014–15. Patients were enrolled within 14 days of initiating EVR and enrollment continued until December 31, 2015. Written informed consent was obtained from all patients before enrollment. Patients who had previously participated in other EVR studies or had exposure to EVR < 3 months prior to enrollment were excluded. Patient medical data were collected using electronic data capture case report forms (CRFs) at 6 months, 1 and 2 years after initiating EVR, or at discontinuation.

### Survey objectives

The survey objective was to assess the safety and effectiveness of EVR in maintenance KTx patients and to identify baseline (at the time of EVR initiation) characteristics influencing renal impairment by observing changes in renal function before and after EVR use. Safety and effectiveness of EVR and changes in renal function from baseline were also assessed in elderly patients (≥ 65 years).

### Sample size determination

A sample size of 200 patients was determined to explore factors influencing renal impairment after EVR initiation. Using findings from a previous retrospective survey in maintenance KTx patients [11], baseline renal function and donor age were considered as prediction factors, each with two categories (i.e., worse/better baseline renal function and younger/older donors). With 200 patients and a 5% alpha level using a chi-squared test, the probabilities of detecting a significant difference between the two categories each for baseline renal function and donor age were 78 and 90%, respectively.

### Survey endpoints and assessments

Safety and effectiveness observation periods were defined as 2 years from EVR initiation until discontinuation or graft loss + 30 days. Safety endpoints included the assessment of serious adverse events (SAEs), adverse drug reactions (ADRs), death, and ADRs by baseline characteristics. SAEs were defined as life-threatening events or death, permanent or significant disability/impairment, congenital abnormality, in-patient hospitalization or prolongation of hospitalization, or medically significant event. Adverse events (AEs) for which a causal relationship with EVR was likely were treated as ADRs. Terminology for AEs was standardized using the Medical Dictionary for Regulatory Activities/Japanese edition, version 21.0. For all AEs, details of causality, action taken, and outcome at each visit until recovery or stabilization were recorded by the investigator. Effectiveness endpoints included the assessment of graft rejection, graft survival, and patient survival rates by baseline characteristics. Graft rejection was clinically diagnosed with/without biopsy and effectiveness rates were defined as the proportion of patients without rejection. ADRs and treatment effectiveness by baseline characteristics were also assessed in elderly patients.

Renal function after initiating EVR was assessed by change in estimated glomerular filtration rate (eGFR; using the Japanese equation [15], international formula [modification of diet in renal disease; MDRD], and serum cystatin C) over time.

Renal impairment at final assessment was defined as percentage decrease in renal function below the 25th percentile eGFR (MDRD) value from EVR initiation, and was assessed by baseline characteristics.

The target trough levels (C_0_) for EVR were determined as 3–8 ng/mL. Mean EVR C_0_ and the proportion of patients within the EVR C_0_ categories (< 3 ng/mL, 3–8 ng/mL, and > 8 ng/mL) were assessed at Months 1, 3, 6, 12, and 24, and at discontinuation.

### Analysis sets

The safety and effectiveness analysis sets were defined as patients with at least one fixed CRF volume in whom none of the exclusion criteria (such as deviations in enrollment/unconfirmed enrollment, patients not receiving EVR, no visit following first dose, unfixed first CRF volume, < 6 months post-transplantation, off-label use, duplicate cases, outside of the contract period, and participation in clinical study of an unapproved drug) were applied.

### Statistical analysis

To assess the influence of baseline characteristics on ADR or effectiveness, Fisher’s exact test (for nominal categorical baseline characteristics) or the Mann–Whitney *U* test (for ordinal categorical baseline characteristics with more than two levels) were performed, with a two-sided significance level of 5%. In testing, “unknown,” “not reported,” and “not evaluable” data were excluded. The Mantel–Haenszel test was performed to adjust the stratified effect of baseline characteristics for which a significant difference (*P* < 0.05) between factors was observed. A factor was suspected to influence ADR or effectiveness if the adjusted analysis showed a significant difference (*P* < 0.05) between factors. Missing values were not imputed and the value from the last assessment point was carried forward for the final assessment. The proportion and odds ratio (95% CI) of patients with renal impairment by baseline characteristics were evaluated in a descriptive manner.

## Results

### Patient demographics and baseline characteristics

The survey enrolled 263 patients from 34 medical institutions, and CRFs for all patients were fixed on August 31, 2018. All 263 patients were included in the safety and effectiveness analysis sets. Demographic and baseline characteristics are presented in Table 1. Mean patient and donor ages were 51.5 ± 13.10 years and 55.8 ± 11.69 years, respectively. Of the 263 patients, 56 (21.29%) were elderly (≥ 65 years). The mean time from transplantation to EVR initiation was 75.7 ± 63.17 months. Decreased renal function (31.56%) was the primary reason for initiating EVR. Baseline eGFR (Japanese equation) was ≤ 60 mL/min/1.73 m^2^ in ~ 80% of patients. EVR was discontinued in 65 (24.71%) patients; AEs being the primary reason for discontinuation in 46 (17.49%) patients.

**Table 1:** Demographic and baseline characteristics (safety analysis set; *N* = 263)

Characteristics	Number of patients, *n* (%)	Characteristics	Number of patients, *n* (%)
Sex		HLA mismatches	
Male	163 (61.98)	< 3	79 (30.04)
Female	100 (38.02)	≥ 3	132 (50.19)
Age (years), mean ± SD	51.5 ± 13.10	Unknown	52 (19.77)
< 50	125 (47.53)	Immunological risk at Tx	
≥ 50 and < 65	82 (31.18)	High risk: ABO-i or PRA( +)	61 (23.19)
≥ 65	56 (21.29)	Normal risk	196 (74.52)
Donor age (years), mean ± SD	55.8 ± 11.69	Unknown	6 (2.28)
< 50	65 (24.71)	Donor type	
≥ 50 and < 65	118 (44.87)	Living	225 (85.55)
≥ 65	55 (20.91)	Cardiac arrest	25 (9.51)
Unknown	25 (9.51)	Brain death	11 (4.18)
Time since Tx (months), mean ± SD	75.7 ± 63.17	Unknown	2 (0.76)
≥ 6 months and < 1 year	48 (18.25)	Reason for initiating EVR	
≥ 1 year and < 5 years	73 (27.76)	Decreased renal function	83 (31.56)
≥ 5 years and < 10 years	88 (33.46)	Malignant tumor	36 (13.69)
≥ 10 years	54 (20.53)	Cardiovascular event	4 (1.52)
Body weight (kg), mean ± SD	58.8 ± 11.69 (*n* = 240)	Arteriosclerosis	29 (11.03)
Height (cm), mean ± SD	163.7 ± 8.85 (*n* = 243)	Cytomegalovirus infection	11 (4.18)
BMI (kg/m^2^), mean ± SD	21.9 ± 3.63 (*n* = 234)	Antimetabolite-related AE	3 (1.14)
< 18.5	39 (14.83)	MMF-related AE	11 (4.18)
≥ 18.5 and < 25.0	160 (60.84)	Other	86 (32.70)
≥ 25.0	35 (13.31)	eGFR (Japanese equation; mL/min/1.73 m^2^)^c^	
Unknown	29 (11.03)	< 30	51 (19.39)
Primary disease leading to KTx^a^		≥ 30 and ≤ 60	161 (61.22)
Chronic glomerulonephritis	80 (30.42)	> 60	48 (18.25)
Focal glomerulosclerosis	10 (3.80)	Unknown	3 (1.14)
IgA nephropathy	51 (19.39)	eGFR (MDRD; mL/min/1.73 m^2^)^c^	
Interstitial nephritis	2 (0.76)	< 30	23 (8.75)
Polycystic kidney	20 (7.60)	≥ 30 and ≤ 60	121 (46.01)
Nephrosclerosis	14 (5.32)	> 60	116 (44.11)
Hypoplastic/dysplastic kidney	4 (1.52)	Unknown	3 (1.14)
Diabetic nephropathy	26 (9.89)	eGFR (serum cystatin C; mL/min/1.73 m^2^)^c^	
Other	60 (22.81)	< 30	22 (8.37)
History of graft rejection^b^		≥ 30 and ≤ 60	80 (30.42)
Cellular rejection	12 (4.56)	> 60	26 (9.89)
ABMR	22 (8.37)	Unknown	135 (51.33)
Relationship with donor		UPCR (g/gCr)^c^	
Blood relative	129 (49.05)	< 0.55	147 (55.89)
Spouse	89 (33.84)	≥ 0.55	19 (7.22)
Other	45 (17.11)	Unknown	97 (36.88)
Unknown	0 (0.0)		

As the safety and effectiveness analysis sets are the same, the composition ratios remain the same for both sets

^a^Primary disease leading to kidney transplantation allowed multiple selections

^b^Within 6 months before the start of EVR treatment

^c^At the start of EVR treatment

*ABMR* antibody-mediated rejection, *ABO-i* ABO incompatible, *AE* adverse event, *BMI* body mass index, *eGFR* estimated glomerular filtration rate, *EVR* everolimus, *HLA* human leukocyte antigen, *IgA* immunoglobulin A, *KTx* kidney transplantation, *MDRD* modification of diet in renal disease, *MMF* mycophenolate mofetil, *PRA* panel reactive antibody, *SD* standard deviation, *Tx* transplantation, *UPCR* urinary protein/creatinine ratio

### Immunosuppression

**Fig. 1:**
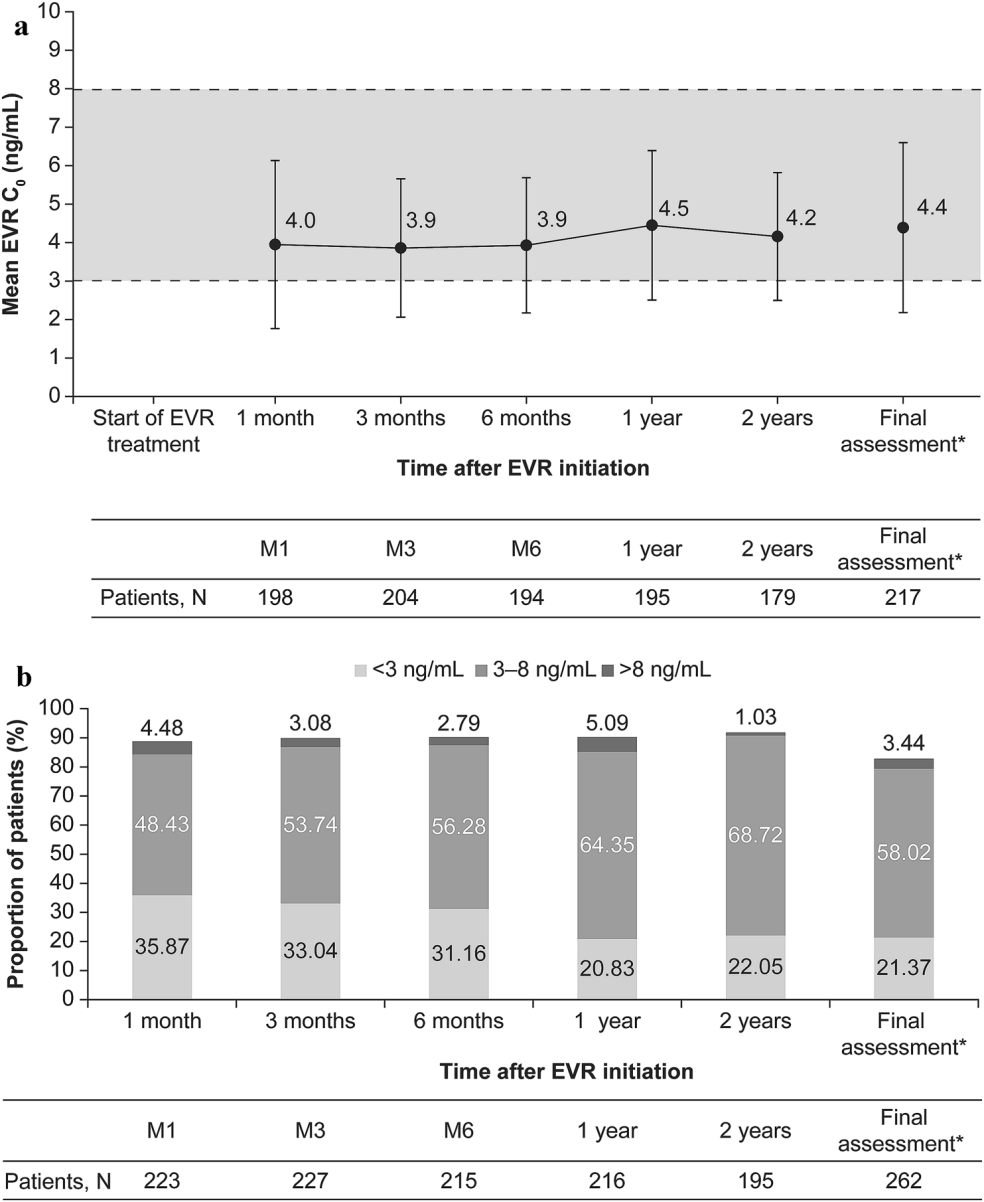
Exposure of everolimus (safety analysis set): **a** Mean (SD) EVR C_0_ over time, **b** Proportion of patients with adherence to the EVR target range (3–8 ng/mL). The shaded box indicates the protocol-defined EVR target C_0_ range (3–8 ng/mL). *Final assessment means at the end of EVR treatment or at discontinuation. *C*_*0*_ trough level, *EVR* everolimus, *M* month, *SD* standard deviation

The mean treatment and observation periods were 613.2 and 644.3 days, respectively. Most patients (72.62%) received EVR for at least 2 years with a mean daily dose of 1.3 mg. Mean EVR C_0_ was within the target range throughout the observation period and was 4.39 ± 2.23 ng/mL at the final assessment (Fig. 1a). Adherence to the target EVR C_0_ was seen in 58.02% of patients (Fig. 1b). During the safety observation period, 182 (69.20%) patients received concomitant tacrolimus and 82 (31.18%) patients received concomitant cyclosporine at least once. By 2 years, the mean daily dose of tacrolimus and cyclosporine was reduced to ~ 79 and ~ 64% of the dose, respectively. The majority of the patients also received mycophenolate mofetil (MMF) (*n* = 224 [85.17%]) and corticosteroids (*n* = 225 [85.55%]) during the survey.

### Safety

The overall incidence of SAEs was 15.97%. Kidney transplant rejection and renal impairment were the most common (1.14% each) SAEs. ADRs occurred in 49.43% of patients, most commonly reported were stomatitis (15.97%), proteinuria (9.89%), hyperlipidemia (5.32%), and peripheral edema (3.80%) (Table 2).

**Table 2:** Incidence rates (≥ 1%) of ADRs by preferred term (safety analysis set; *N* = 263)

Incidence of ADRs *n*/*N* (%)	130/263 (49.43)
Type of ADR	Incidence, *n* (%)
Nasopharyngitis	3 (1.14)
Kidney transplant rejection	3 (1.14)
Dyslipidemia	6 (2.28)
Lipid metabolism disorder	3 (1.14)
Hyperlipidemia	14 (5.32)
Diarrhea	3 (1.14)
Stomatitis	42 (15.97)
Rash	4 (1.52)
Proteinuria	26 (9.89)
Renal impairment	5 (1.90)
Concomitant disease aggravated	3 (1.14)
Peripheral edema	10 (3.80)
Albumin urine present	7 (2.66)
Blood creatinine increased	3 *(*1.14)
Protein urine	3 (1.14)
Protein urine present	5 (1.90)

*ADR* adverse drug reaction

Two deaths were reported during the survey. One patient (76 years) died from gastric cancer. Onset was seen on Day 28 post-EVR initiation, and the time from transplantation to EVR initiation was 4.2 years. Another patient (72 years) died from subarachnoid hemorrhage with an onset of 40 days after EVR initiation. The time post-transplantation to EVR initiation was 6.2 years. Both deaths were reported to be unrelated to EVR treatment.

While investigating the incidence of ADRs by baseline characteristics (Table 3), history of antibody-mediated rejection (ABMR), donor type, and reasons for initiating EVR were identified as contributing factors. The frequency of ADRs was higher in patients with versus without a history of ABMR (77.27 versus 47.08%; *P* = 0.0074). Most ADRs occurred in patients receiving an allograft from brain-death donors (63.64%), followed by living (51.11%) and cardiac arrest (28.0%) donors (*P* = 0.0494). However, adjusted analysis for ABMR and donor type categories showed no significant differences, suggesting that these findings may be due to confounders. The incidence of ADRs (*P* = 0.0010) by reasons for initiating EVR is provided in Table 3. As the adjusted analysis also found significant differences, reasons for initiating EVR should be considered as an influencing factor. The incidence of ADRs in elderly patients (41.07%) was numerically lower than in non-elderly patients (51.69%).

**Table 3:**
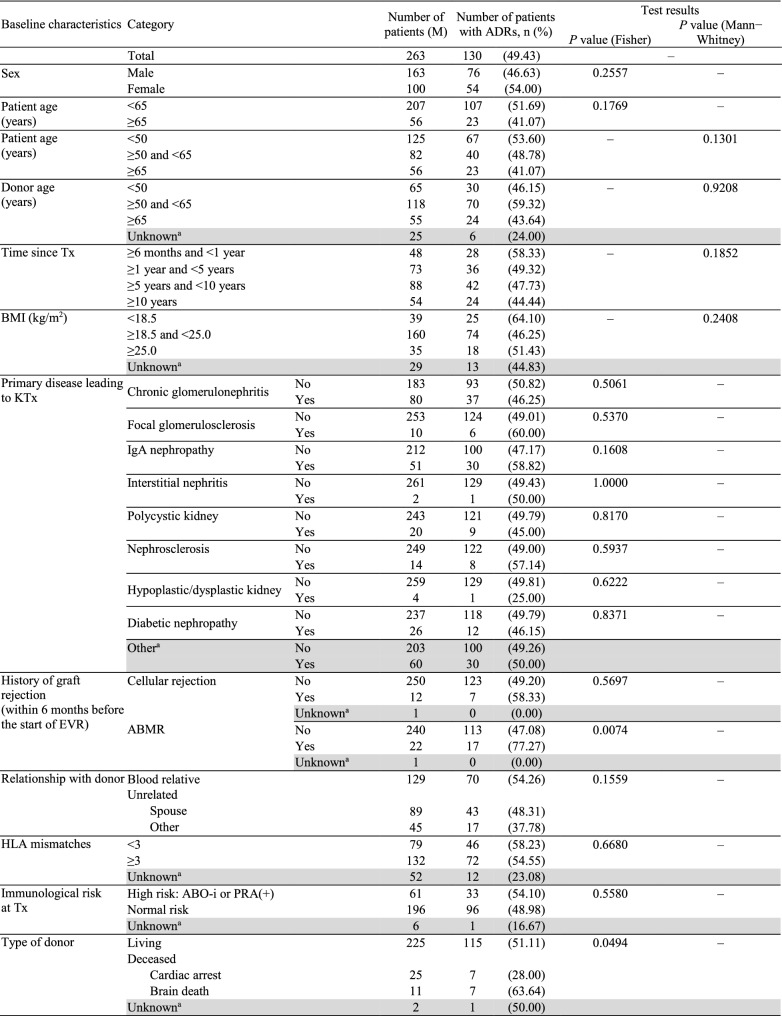

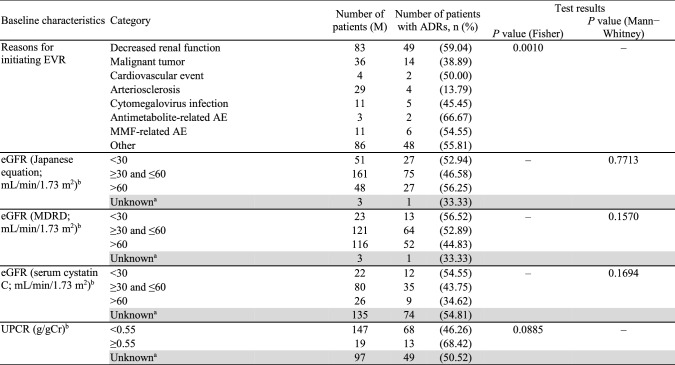
Incidence of ADRs by baseline characteristics (safety analysis set)

^a^Shaded categories were not considered for tests

^b^At the start of EVR treatment

*ABMR* antibody-mediated rejection, *ABO-i* ABO incompatible, *ADR* adverse drug reaction, *AE* adverse event, *BMI* body mass index, *eGFR* estimated glomerular filtration rate, *EVR* everolimus, *HLA* human leukocyte antigen, *IgA* immunoglobulin A, *KTx* kidney transplantation, *MDRD* modification of diet in renal disease, *MMF* mycophenolate mofetil, *PRA* panel reactive antibody, *Tx* transplantation, *UPCR* urinary protein/creatinine ratio

### Effectiveness

Overall incidence of graft rejection was low (6.84%) in this population. In total, 19 events were reported in 18 patients (Table S1). The 2-year graft survival rate was 95.82% and the patient survival rate was 99.24%. Effectiveness analysis by baseline characteristics is presented in Table 4.

**Table 4:**
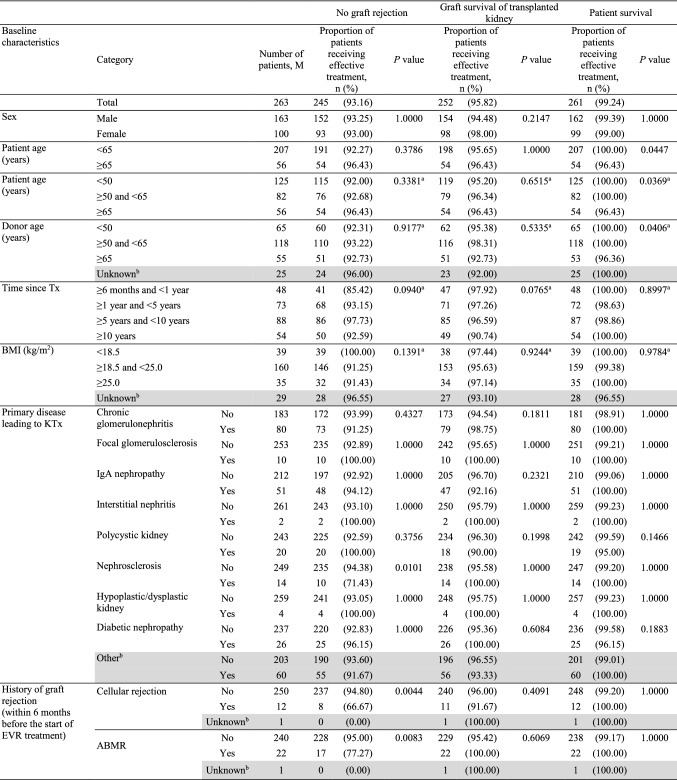

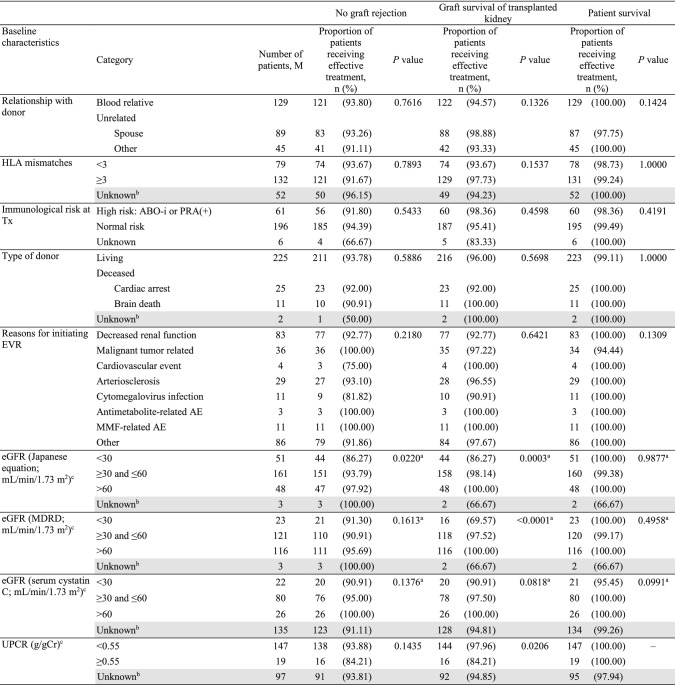
Effectiveness analysis by baseline characteristics (effectiveness analysis set)

^a^*P* values calculated using the Mann − Whitney *U* test. All other *P* values are calculated using Fisher’s exact test

^b^Shaded categories were not considered for tests

^c^At the start of EVR treatment

*ABMR* antibody-mediated rejection, *ABO-i* ABO incompatible, *AE* adverse event, *BMI* body mass index, *eGFR* estimated glomerular filtration rate, *EVR* everolimus, *HLA* human leukocyte antigen, *IgA* immunoglobulin A, *KTx* kidney transplantation, *MDRD* modification of diet in renal disease, *MMF* mycophenolate mofetil, *PRA* panel reactive antibody, *Tx* transplantation, *UPCR* urinary protein/creatinine ratio

#### Graft rejection

Baseline characteristics such as nephrosclerosis, history of cellular rejection and ABMR, and eGFR (Japanese equation) were found to influence graft rejection (Table 4). Patients with nephrosclerosis before transplantation showed a lower rate of effectiveness (71.43%) compared to patients without nephrosclerosis (94.38%; *P* = 0.0101). The rates of effectiveness were lower in patients with versus without a history of cellular rejection (66.67 versus 94.80%; *P* = 0.0044) and in patients with versus without a history of ABMR (77.27 versus 95.00%; *P* = 0.0083). As the adjusted analysis also found a significant difference, the influence of nephrosclerosis and history of both cellular rejection and ABMR could not be eliminated. The rates of effectiveness were higher in patients within the eGFR categories (Japanese equation) of ≥ 30 and ≤ 60 mL/min/1.73 m^2^, and > 60 mL/min/1.73 m^2^ (*P* = 0.0220). These differences by eGFR category may be due to confounding factors, as the adjusted analysis showed no significant differences. Effectiveness rates were comparable between elderly and non-elderly patients (96.43 versus 92.27%; *P* = 0.3786).

#### Graft survival

Graft survival rates were influenced by baseline eGFR (Japanese equation and MDRD) and urinary protein/creatinine ratio (UPCR). Patients with lower baseline eGFR showed lower graft survival rates compared to patients with higher eGFR (> 60 mL/min/1.73 m^2^; *P* = 0.0003 for eGFR [Japanese equation] and *P* < 0.0001 for eGFR [MDRD]). Baseline UPCR of < 0.55 g/gCr showed a higher 2-year graft survival rate (97.96%) versus UPCR ≥ 0.55 g/gCr (84.21%; *P* = 0.0206) (Table 4). As adjusted analysis failed to show significant differences, the influence of baseline eGFR (Japanese equation) and UPCR could be due to confounding factors. Graft survival rates were comparable between elderly and non-elderly patients (96.43 versus 95.65%; *P* = 1.0000).

#### Patient survival

Patient survival rates were associated with baseline characteristics such as patient and donor age. The survival rates were lower in elderly (96.43%) versus non-elderly (100.00%; *P* = 0.0447) patients (Table 4). Adjusted analysis for patient age also showed significant differences. The two deaths reported during the survey due to gastric cancer and subarachnoid hemorrhage, respectively, were seen in elderly patients. However, the causal relationship between these AEs leading to death and EVR was eliminated for each instance; therefore, an advanced age was not considered to directly affect survival in these patients. Patient survival rates by donor age categories (*P* = 0.0406) are provided in Table 4. The adjusted analysis showed that the influence of donor age on patient survival could be due to confounding factors.

### Renal function

**Fig. 2:**
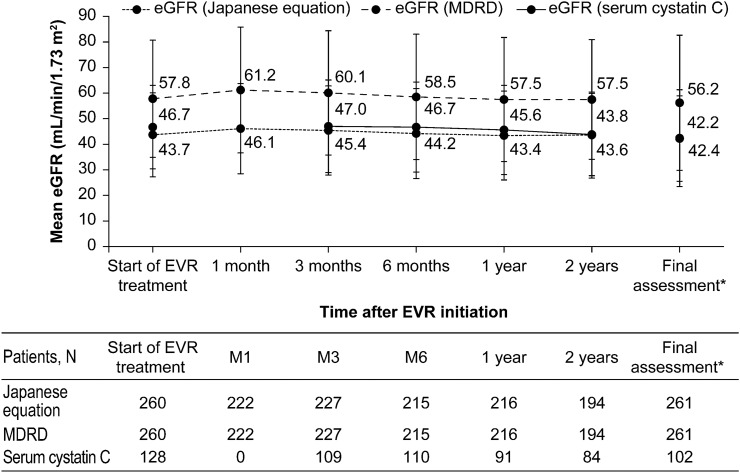
Mean (SD) change in eGFR over time (safety analysis set). *Final assessment means at the end of EVR treatment or at discontinuation. *eGFR* estimated glomerular filtration rate, *EVR* everolimus, *M* month, *MDRD* modification of diet in renal disease, *SD* standard deviation

**Fig. 3:**
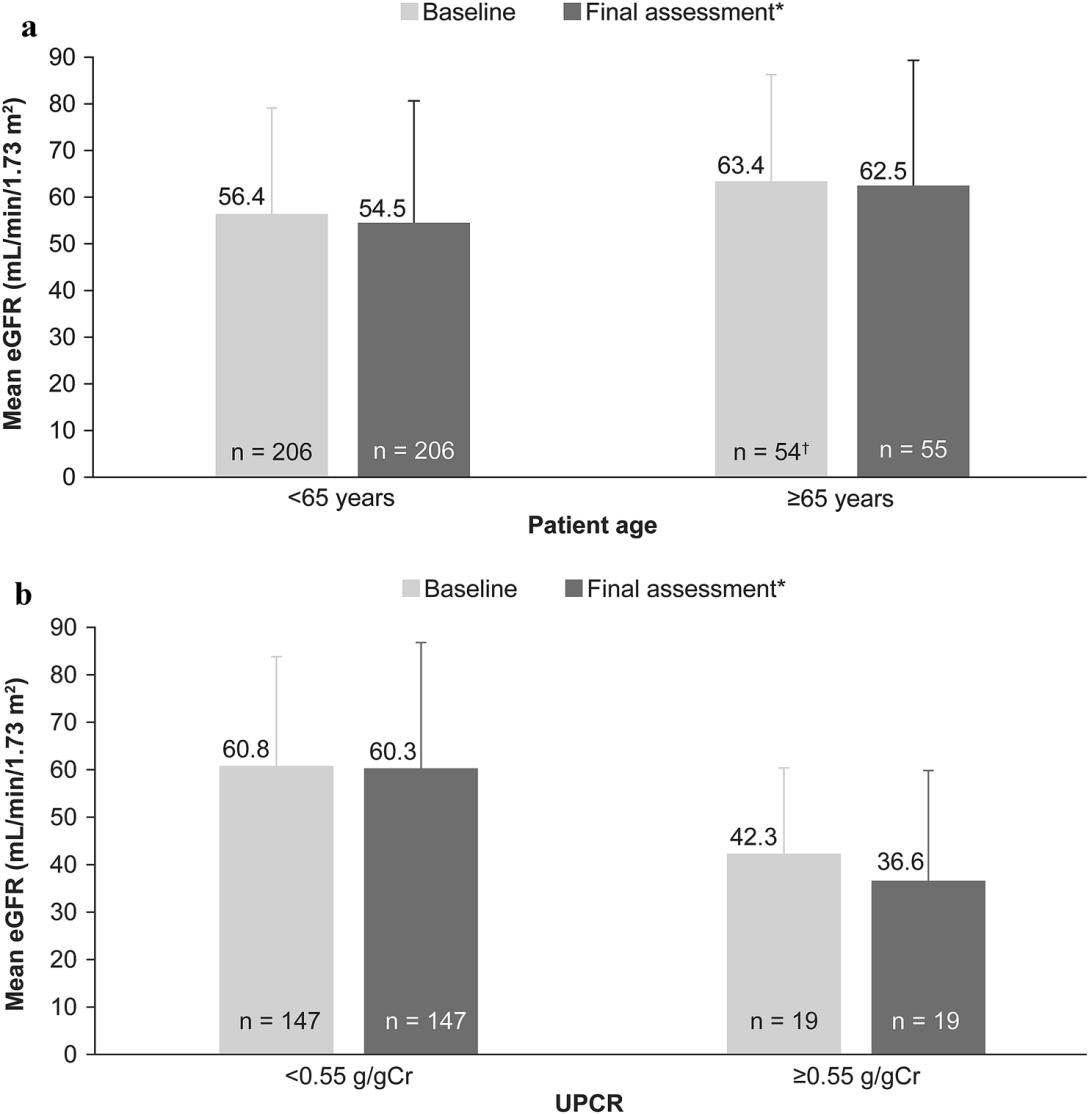
Mean (SD) change in eGFR (MDRD; safety analysis set): **a** By patient age (< 65 years vs ≥ 65 years), **b** By UPCR categories (< 0.55 g/gCr vs ≥ 0.55 g/gCr). *Final assessment means at the end of EVR treatment or at discontinuation; ^†^Changes over time in the renal function data during the observation period were calculated for patients who had data at each measurement time point out of the 263 patients in the safety analysis set. *eGFR* estimated glomerular filtration rate, *EVR* everolimus, *MDRD* modification of diet in renal disease, *SD* standard deviation, *UPCR* urinary protein/creatinine ratio

Baseline mean eGFR values were maintained until 2 years after the treatment or the final assessment (Fig. 2). For patients aged < 65 years and ≥ 65 years, mean eGFR (MDRD) at baseline and final assessment were comparable (Fig. 3a). Among patients with baseline UPCR ≥ 0.55 g/gCr, mean eGFR (MDRD) at the final assessment was numerically lower compared to baseline (Fig. 3b).

The proportion of patients with renal impairment (eGFR [MDRD]) at the final assessment by baseline characteristics is presented in Table 5 (renal impairment assessments by eGFR [Japanese equation] and eGFR [serum cystatin C] are given in Table S2 and S3, respectively). Baseline factors such as patient and donor age, eGFR, and UPCR were found to influence renal impairment after EVR treatment. Incidence of renal impairment was lower in the patient age group of ≥ 65 years (16.98%) versus < 50 years (31.71%). In patients with donors aged ≥ 65 years, the incidence of renal impairment was higher (38.89%) versus donors aged < 50 years (20.31%). Irrespective of the formula used for measuring eGFR, more patients with baseline eGFR < 30 mL/min/1.73 m^2^ showed renal impairment at the final assessment. Of 23 patients with baseline eGFR (MDRD) < 30 mL/min/1.73 m^2^, 12 (52.17%) showed renal impairment at the final assessment. Similarly, incidence (52.63%) of renal impairment was higher in patients with baseline UPCR ≥ 0.55 g/gCr.

**Table 5:**
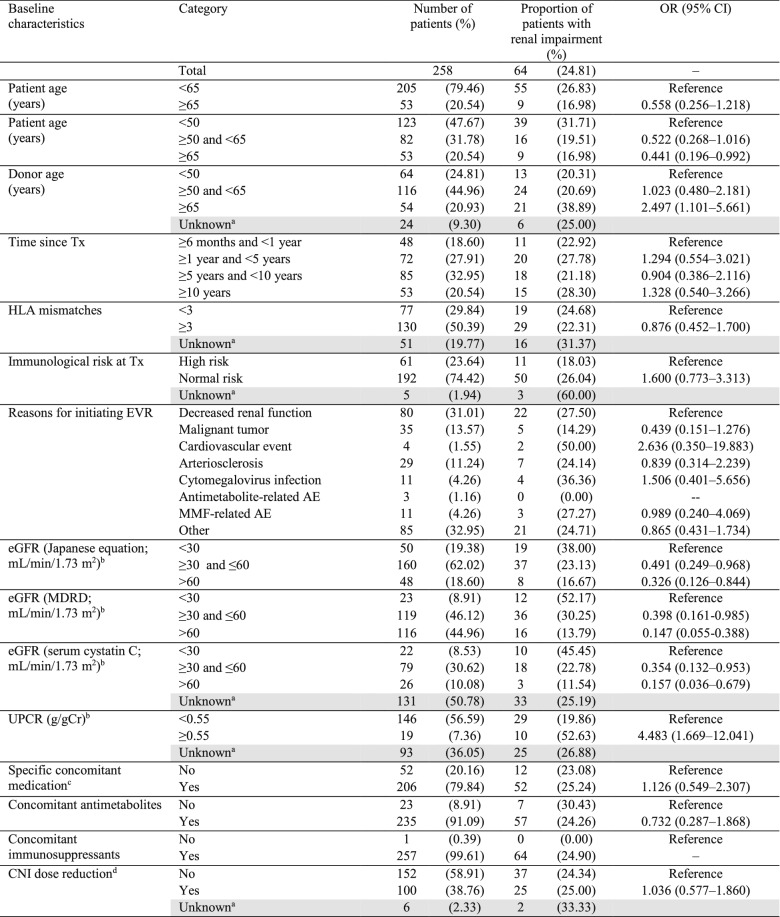
Proportion of patients with renal impairment by baseline characteristics: percentage decrease in renal function below the 25th percentile eGFR (MDRD)

^a^Shaded categories were not considered for tests

^b^At the start of EVR treatment

^c^Angiotensin II receptor antagonists, angiotensin-converting enzyme inhibitors, treatment drugs for dyslipidemia, and treatment drugs for diabetes mellitus including insulin

^d^Patients with CNI dose reduction were defined as those in whom the dosage of CNIs was reduced by ≥ 30% relative to the dose at the start of treatment at ≥ 2 time points out of all assessment points

*AE* adverse event, *CI* confidence interval, *CNI* calcineurin inhibitor, *eGFR* estimated glomerular filtration rate, *EVR* everolimus, *HLA* human leukocyte antigen, *MDRD* modification of diet in renal disease, *MMF* mycophenolate mofetil, *OR* odds ratio, *Tx* transplantation, *UPCR* urinary protein/creatinine ratio

## Discussion

This large PMS study in Japan reported real-world use of EVR in 263 adults, maintenance KTx patients (with 225 living donor transplants), and identified baseline characteristics influencing the subsequent renal impairment. AEs were the primary reason for EVR discontinuation and the most common ADRs reported were stomatitis, proteinuria, hyperlipidemia, and peripheral edema. These safety findings are consistent with those previously reported in the literature for EVR [9–12]. In our survey, with EVR initiation, the mean daily dose of tacrolimus and cyclosporine was reduced to ~ 79 and ~ 64% by 2 years, respectively. Even though blood CNI concentrations were measured at various time points, mean CNI C_0_ levels were not reported due to a lack of data on the timing of blood sampling. However, post-hoc analysis (data not shown) showed an overall decline in blood CNI levels over the course of EVR treatment.

Late conversion (≥ 3 years after transplantation) to EVR has shown to impact renal function and graft survival [11]. Although the overall graft survival rate (95.82%) at 2 years was high in our survey, low rates were seen in patients with baseline eGFR (MDRD) < 30 mL/min/1.73 m^2^ and UPCR ≥ 0.55 g/gCr. Similar results were observed in the ASCERTAIN study (assessing the effect of late-conversion [mean 5.6 years post-transplant] to EVR with CNI elimination/minimization in maintenance KTx patients with renal impairment at baseline [defined as GFR 30–70 mL/min]), where overall, 93.3% of patients receiving EVR survived with a functioning graft at Month (M) 24 [12]. This suggests that longer follow-up (beyond 2 years) is needed to conclude the clinical benefits of EVR.

In the ASCERTAIN study, renal function was stable in all treatment groups at M24. Although no overall renal benefit was seen with EVR-based regimen, post-hoc analyses showed that the increase in measured glomerular filtration rate (mGFR) was higher in patients with baseline creatinine clearance (CrCl) > 50 mL/min in the CNI elimination group versus the control group (*P* = 0.017) [12]. In a retrospective study in KTx patients, improvement in renal function was statistically significant at 1-year in patients who were converted (median 5.8 years post-transplant) to EVR with baseline CrCl ≥ 40 mL/min and proteinuria < 550 mg/day (*P* = 0.005) [11]. Consistent with these findings, baseline mean eGFR values were maintained during our survey. However, renal impairment at final assessment was higher in patients with baseline eGFR (MDRD) < 30 mL/min/1.73 m^2^ and UPCR ≥ 0.55 g/gCr. This suggests that patients with good baseline renal function may benefit from an EVR-based regimen. In contrast, Nojima et al. reported significant improvement in renal function at 1-year in Japanese KTx patients, including patients with low baseline eGFR (< 30 mL/min) converted to EVR-based regimen at a mean 7.4 years post-transplant. This could be due to the low CNI C_0_ levels observed in this study [16].

The lower incidence of renal impairment in elderly patients versus patients aged < 50 years could be due to a high proportion of (i) elderly patients who received a graft from donors aged < 65 years and (ii) patients aged < 50 years who received a graft from donors aged ≥ 50 years. As renal function declines with age [11], graft function may have been impaired at transplantation in patients < 50 years who received a graft from elderly donors. However, adjusted analysis with confounders was not performed to confirm this finding.

The low graft rejection rate in our survey could be because most patients received EVR + CNI + MMF + corticosteroids therapy. Despite the significant difference in the effectiveness rates between patients with versus without nephrosclerosis, clinical factors potentially associated with the observed difference could not be identified in this survey setting due to a small number of patients with nephrosclerosis.

Randomized studies with early EVR initiation (< 6 months post-transplant) have shown clinical benefits of using EVR. In the ZEUS study, conversion to EVR with cyclosporine elimination showed significant improvement in renal function up to 5 years (*P* < 0.001), while maintaining efficacy and safety [17, 18]. Similarly, conversion to EVR with cyclosporine elimination was associated with a significant increase in mGFR (*P* = 0.012) at M12 in the CENTRAL study [19] and a significant increase in eGFR (*P* < 0.001) up to M24 in the ELEVATE study [20]. Results from the largest study in de novo KTx patients (TRANSFORM) with 50% living donor transplants showed comparable antirejection efficacy, stable renal function, and low incidence of de novo donor-specific antibodies (dnDSA) and viral infections with EVR-based regimen at M24 [9]. The clinical benefit of EVR was also evident in de novo Japanese KTx patients in the 12-month A1202 study, where EVR + reduced-exposure cyclosporine (EVR + rCsA) group showed numerically higher median eGFR values (58 mL/min/1.73 m^2^ versus 55.25 mL/min/1.73 m^2^; *P* = 0.063) and comparable safety versus MMF + standard-exposure cyclosporine (MMF + sCsA) group. The graft survival rate at M12 was 100% in both the treatment groups [21]. Moreover, when participants (*N *= 24) from this study were followed-up at 10 years, the graft survival rate was maintained in the EVR + rCsA (100%) group but was reduced in the MMF + sCsA (90.9%) group. In addition, dnDSA-free survival was significantly better in the EVR + rCsA group [22]. Although these results are from a small population, early EVR initiation showed better clinical outcomes. More robust clinical evidence is needed to conclude the long-term benefits of EVR in Japanese KTx patients.

The main limitations of this survey were lack of a comparator arm to conclude the clinical benefits of EVR and a limited follow-up period of 2 years. In addition, protocol-defined criteria for confirmation of rejection and graft survival were not applicable. However, the survey provided useful insights into the safety and effectiveness of EVR use in Japanese KTx patients in a real-world setting.

In conclusion, this survey showed that EVR initiation can facilitate the reduction of mean daily doses of tacrolimus and cyclosporine to ~ 79 and ~ 64% by 2 years, respectively. Although overall patient and graft survival rates at 2 years were high, graft survival rates were affected by baseline eGFR and UPCR values. Renal impairment was higher in patients with poor baseline eGFR and UPCR. Thus, early EVR initiation (< 6 months post-transplant) may be considered in maintenance KTx patients to prevent renal function deterioration. No new safety concerns for EVR use in Japanese maintenance KTx patients were identified during the survey.

## Supplementary Information

Below is the link to the electronic supplementary material.

Supplementary file1 (DOCX 30 KB)

Supplementary file2 (DOCX 35 KB)

Supplementary file3 (DOCX 35 KB)

## Abbreviations

ABMR: Antibody-mediated rejection
ADR: Adverse drug reaction
AE: Adverse event
C_0_: Trough level
CNI: Calcineurin inhibitor
CRF: Case report form
DSA: Donor-specific antibodies
eGFR: Estimated glomerular filtration rate
EVR: Everolimus
MDRD: Modification of diet in renal disease
mGFR: Measured glomerular filtration rate
MMF: Mycophenolate mofetil
rCsA: Reduced-exposure cyclosporine
SAE: Serious adverse event
SD: Standard deviation
sCsA: Standard-exposure cyclosporine
UPCR: Urinary protein/creatinine ratio
