# Supplementary material for: Safety and effectiveness of everolimus in maintenance kidney transplant patients in the real-world setting: results from a 2-year post-marketing surveillance study in Japan
Source: Clin Exp Nephrol. 2021 Feb 11;25(6):660–73. doi: 10.1007/s10157-021-02024-9 (PMC8106613; doi:10.1007/s10157-021-02024-9)
Supplement: Supplementary file 1 — Supplementary file1 (DOCX 30 KB) [file 10157_2021_2024_MOESM1_ESM.docx]

**Table S1.** Graft rejection (effectiveness analysis set)

| Patient no. | Recipient age  (years) | Donor age  (years) | Time post-Tx at EVR initiation (years) | Time to event after EVR initiation  (days) | Graft rejection type | Treatment | Influence on graft | Biopsy performed | Causal relationship with EVR | Type or classification | |
| --- | --- | --- | --- | --- | --- | --- | --- | --- | --- | --- | --- |
| 1 | 45 | 40 | 0.58 | 131 | ABMR | Antibody therapy/plasma exchange | Others | Yes | NR | NA | |
| 2 | 27 | 49 | 2.07 | 337 | Cellular rejection | Steroid pulse therapy/antibody therapy/immunoglobulin | Possible graft loss | Yes | NR | NA | |
| 3 | 42 | 61 | 4.69 | 330 | Other (ABMR + cellular rejection) | Steroid pulse therapy | Others | No | NR | Original disease or complication | |
| 4 | 54 | 67 | 2.63 | 162 | Other (chronic active ABMR + cellular rejection) | Steroid pulse therapy | Others | No | NR | Original disease or complication | |
|  |  |  |  | 598 | ABMR | Steroid pulse therapy | Others | No | NR | Original disease or complication | |
| 5 | 38 | Unknown | 11.15 | 257 | Other  (borderline) | No action (observation of the course only) | Others | Yes | NR | Original disease or complication | |
| 6 | 38 | 63 | 11.27 | 182 | ABMR | Discontinuation of EVR/steroid pulse therapy/plasma exchange | Graft loss | Yes | NR | NA | |
| 7 | 55 | 55 | 0.77 | 84 | Cellular rejection | Steroid pulse therapy | Others | Yes | NR | Concomitant medication (CsA) | |
| 8 | 68 | 63 | 5.25 | 177 | ABMR | Steroid pulse therapy | Others | Yes | NR | NA | |
| 9 | 58 | 45 | 10.82 | 351 | ABMR | No action (observation of the course only) | Others | Yes | NR | NA | |
| 10 | 47 | 67 | 1.33 | 236 | ABMR | Change of EVR dose | Possible graft loss | Yes | NR | NA | |
| 11 | 42 | 56 | 16.41 | 261 | ABMR | Change of EVR dose/steroid pulse therapy | Possible graft loss | Yes | NR | Original disease or complication | |
| 12 | 50 | 77 | 0.52 | 168 | ABMR | Steroid pulse/antibody therapy | Others | Yes | R | Original disease or complication | |
| 13 | 63 | 59 | 1.39 | 151 | Cellular rejection | Steroid pulse therapy/other (increasing CsA) | Others | Yes | R | Concomitant medication (CsA reduction) | |
| 14 | 40 | 46 | 0.64 | 267 | Cellular rejection | Steroid pulse therapy | Others | Yes | NR | NA | |
| 15 | 67 | 62 | 0.53 | 196 | ABMR | No action (observation of the course only) | Others | Yes | NR | Original disease or complication | |
| 16 | 45 | 65 | 5.21 | 1 | ABMR | No action (observation of the course only) | Graft loss | Yes | NR | NA | |
| 17 | 58 | 61 | 0.56 | 531 | Other  (BKV Stage B) | Steroid pulse therapy | Possible graft loss | Yes | NR | Original disease or complication | |
| 18 | 30 | 42 | 0.55 | 245 | Cellular rejection | Steroid pulse therapy | Others | Yes | R | concomitant medication (TAC/MMF) | |
| ABMR, antibody-mediated rejection; BKV, BK virus; CsA, cyclosporine A; EVR, everolimus; MMF, mycophenolate mofetil; NA, not available; NR, not related; R, related; TAC, tacrolimus; Tx, transplantation | | | | | | | | | | |  |
