# Supplementary material for: Safety and effectiveness of everolimus in maintenance kidney transplant patients in the real-world setting: results from a 2-year post-marketing surveillance study in Japan
Source: Clin Exp Nephrol. 2021 Feb 11;25(6):660–73. doi: 10.1007/s10157-021-02024-9 (PMC8106613; doi:10.1007/s10157-021-02024-9)
Supplement: Supplementary file 2 — Supplementary file2 (DOCX 35 KB) [file 10157_2021_2024_MOESM2_ESM.docx]

**Table S2.** Proportion of patients with renal impairment by baseline characteristics: Percentage decrease in renal function below the 25^th^ percentile eGFR (Japanese equation)

| Baseline characteristics | Category | | Number of patients (%) | | Proportion of patients with renal impairment (%) | | OR (95% CI) |
| --- | --- | --- | --- | --- | --- | --- | --- |
| Total | | | 258 | | 64 | (24.81) | – |
| Patient age  (years) | <65 | | 205 | (79.46) | 55 | (26.83) | Reference |
|  | ≥65 | | 53 | (20.54) | 9 | (16.98) | 0.558 (0.256–1.218) |
| Patient age  (years) | <50 | | 123 | (47.67) | 39 | (31.71) | Reference |
|  | ≥50 and <65 | | 82 | (31.78) | 16 | (19.51) | 0.522 (0.268–1.016) |
|  | ≥65 | | 53 | (20.54) | 9 | (16.98) | 0.441 (0.196–0.992) |
| Donor age  (years) | <50 | | 64 | (24.81) | 13 | (20.31) | Reference |
|  | ≥50 and <65 | | 116 | (44.96) | 24 | (20.69) | 1.023 (0.480–2.181) |
|  | ≥65 | | 54 | (20.93) | 21 | (38.89) | 2.497 (1.101–5.661) |
|  | Unknown^a^ | | 24 | (9.30) | 6 | (25.00) |  |
| Time since Tx | ≥6 months and <1 year | | 48 | (18.60) | 11 | (22.92) | Reference |
|  | ≥1 year and <5 years | | 72 | (27.91) | 20 | (27.78) | 1.294 (0.554–3.021) |
|  | ≥5 years and <10 years | | 85 | (32.95) | 18 | (21.18) | 0.904 (0.386–2.116) |
|  | ≥10 years | | 53 | (20.54) | 15 | (28.30) | 1.328 (0.540–3.266) |
| HLA mismatches | <3 | | 77 | (29.84) | 19 | (24.68) | Reference |
|  | ≥3 | | 130 | (50.39) | 29 | (22.31) | 0.876 (0.452–1.700) |
|  | Unknown^a^ | | 51 | (19.77) | 16 | (31.37) |  |
| Immunological risk at Tx | High risk | | 61 | (23.64) | 11 | (18.03) | Reference |
|  | Normal risk | | 192 | (74.42) | 50 | (26.04) | 1.600 (0.773–3.313) |
|  | Unknown^a^ | | 5 | (1.94) | 3 | (60.00) |  |
| Reasons for initiating EVR | Decreased renal function | | 80 | (31.01) | 22 | (27.50) | Reference |
|  | Malignant tumor | | 35 | (13.57) | 5 | (14.29) | 0.439 (0.151–1.276) |
|  | Cardiovascular event | | 4 | (1.55) | 2 | (50.00) | 2.636 (0.350–19.883) |
|  | Arteriosclerosis | | 29 | (11.24) | 7 | (24.14) | 0.839 (0.314–2.239) |
|  | Cytomegalovirus infection | | 11 | (4.26) | 4 | (36.36) | 1.506 (0.401–5.656) |
|  | Antimetabolite-related AE | | 3 | (1.16) | 0 | (0.00) | – |
|  | MMF-related AE | | 11 | (4.26) | 3 | (27.27) | 0.989 (0.240–4.069) |
|  | Other | | 85 | (32.95) | 21 | (24.71) | 0.865 (0.431–1.734) |
| eGFR (Japanese equation; mL/min/1.73 m^2^)^b^ | <30 | | 50 | (19.38) | 19 | (38.00) | Reference |
|  | ≥30 and ≤60 | | 160 | (62.02) | 37 | (23.13) | 0.491 (0.249–0.968) |
|  | >60 | | 48 | (18.60) | 8 | (16.67) | 0.326 (0.126–0.844) |
| eGFR (MDRD; mL/min/1.73 m^2^)^b^ | <30 | | 23 | (8.91) | 12 | (52.17) | Reference |
|  | ≥30 and ≤60 | | 119 | (46.12) | 36 | (30.25) | 0.398 (0.161–0.985) |
|  | >60 | | 116 | (44.96) | 16 | (13.79) | 0.147 (0.055–0.388) |
| eGFR (serum cystatin C; mL/min/1.73 m^2^)^b^ | <30 | | 22 | (8.53) | 10 | (45.45) | Reference |
|  | ≥30 and ≤60 | | 79 | (30.62) | 18 | (22.78) | 0.354 (0.132–0.953) |
|  | >60 | | 26 | (10.08) | 3 | (11.54) | 0.157 (0.036–0.679) |
|  | Unknown^a^ |  | 131 | (50.78) | 33 | (25.19) |  |
| UPCR^b^ (g/gCr) | <0.55 | | 146 | (56.59) | 29 | (19.86) | Reference |
|  | ≥0.55 | | 19 | (7.36) | 10 | (52.63) | 4.483 (1.669–12.041) |
|  | Unknown^a^ |  | 93 | (36.05) | 25 | (26.88) |  |
| Specific concomitant medication^c^ | No |  | 52 | (20.16) | 12 | (23.08) | Reference |
|  | Yes |  | 206 | (79.84) | 52 | (25.24) | 1.126 (0.549–2.307) |
| Concomitant antimetabolites | No |  | 23 | (8.91) | 7 | (30.43) | Reference |
|  | Yes |  | 235 | (91.09) | 57 | (24.26) | 0.732 (0.287–1.868) |
| Concomitant immunosuppressants | No |  | 1 | (0.39) | 0 | (0.00) | Reference |
|  | Yes |  | 257 | (99.61) | 64 | (24.90) | – |
| CNI dose reduction^d^ | No |  | 152 | (58.91) | 37 | (24.34) | Reference |
|  | Yes |  | 100 | (38.76) | 25 | (25.00) | 1.036 (0.577–1.860) |
|  | Unknown^a^ |  | 6 | (2.33) | 2 | (33.33) |  |
| ^a^Shaded categories were not considered for tests; ^b^At the start of EVR treatment; ^c^Angiotensin II receptor antagonists, angiotensin-converting enzyme inhibitors, treatment drugs for dyslipidemia, and treatment drugs for diabetes mellitus including insulin; ^d^Patients with CNI dose reduction were defined as those in whom the dosage of CNIs was reduced by ≥ 30% relative to the dose at the start of treatment at ≥2 time points out of all assessment points.  AE, adverse event; CI, confidence interval; CNI, calcineurin inhibitor; eGFR, estimated glomerular filtration rate; EVR, everolimus; HLA, human leukocyte antigen; MDRD, modification of diet in renal disease; MMF, mycophenolate mofetil; OR, odds ratio; Tx, transplantation; UPCR, urinary protein/creatinine ratio | | | | | | | |
