# Supplementary material for: Safety and effectiveness of everolimus in maintenance kidney transplant patients in the real-world setting: results from a 2-year post-marketing surveillance study in Japan
Source: Clin Exp Nephrol. 2021 Feb 11;25(6):660–73. doi: 10.1007/s10157-021-02024-9 (PMC8106613; doi:10.1007/s10157-021-02024-9)
Supplement: Supplementary file 3 — Supplementary file3 (DOCX 35 KB) [file 10157_2021_2024_MOESM3_ESM.docx]

**Table S3.** Proportion of patients with renal impairment by baseline characteristics: Percentage decrease in renal function below the 25^th^ percentile eGFR (serum cystatin C)

| Baseline  characteristics | Category | | Number of patients (%) | | Proportion of patients with renal impairment (%) | | OR (95% CI) |
| --- | --- | --- | --- | --- | --- | --- | --- |
| Total | | | 93 | | 23 | (24.73) | – |
| Patient age  (years) | <65 | | 72 | (77.42) | 18 | (25.00) | Reference |
|  | ≥65 | | 21 | (22.58) | 5 | (23.81) | 0.938 (0.301–2.923) |
| Patient age  (years) | <50 | | 47 | (50.54) | 13 | (27.66) | Reference |
|  | ≥50 and <65 | | 25 | (26.88) | 5 | (20.00) | 0.654 (0.203–2.107) |
|  | ≥65 | | 21 | (22.58) | 5 | (23.81) | 0.817 (0.249–2.687) |
| Donor age  (years) | <50 | | 23 | (24.73) | 4 | (17.39) | Reference |
|  | ≥50 and <65 | | 42 | (45.16) | 10 | (23.81) | 1.484 (0.408–5.398) |
|  | ≥65 | | 17 | (18.28) | 6 | (35.29) | 2.591 (0.598–11.234) |
|  | Unknown^a^ |  | 11 | (11.83) | 3 | (27.27) |  |
| Time since Tx | ≥6 months and <1 year | | 12 | (12.90) | 5 | (41.67) | Reference |
|  | ≥1 year and <5 years | | 33 | (35.48) | 8 | (24.24) | 0.448 (0.111–1.811) |
|  | ≥5 years and <10 years | | 28 | (30.11) | 7 | (25.00) | 0.467 (0.112–1.953) |
|  | ≥10 years | | 20 | (21.51) | 3 | (15.00) | 0.247 (0.046–1.326) |
| HLA mismatches | <3 | | 27 | (29.03) | 8 | (29.63) | Reference |
|  | ≥3 | | 39 | (41.94) | 10 | (25.64) | 0.819 (0.274–2.448) |
|  | Unknown^a^ |  | 27 | (29.03) | 5 | (18.52) |  |
| Immunological risk at Tx | High risk | | 23 | (24.73) | 7 | (30.43) | Reference |
|  | Normal risk | | 68 | (73.12) | 15 | (22.06) | 0.647 (0.225–1.862) |
|  | Unknown^a^ |  | 2 | (2.15) | 1 | (50.00) |  |
| Reasons for initiating EVR | Decreased renal function | | 30 | (32.26) | 6 | (20.00) | Reference |
|  | Malignant tumor | | 19 | (20.43) | 3 | (15.79) | 0.750 (0.163–3.441) |
|  | Cardiovascular event | | 2 | (2.15) | 1 | (50.00) | 4.000 (0.217–73.618) |
|  | Arteriosclerosis | | 16 | (17.20) | 4 | (25.00) | 1.333 (0.315–5.642) |
|  | Cytomegalovirus infection | | 4 | (4.30) | 2 | (50.00) | 4.000 (0.464–34.493) |
|  | Antimetabolite-related AE | | 2 | (2.15) | 0 | (0.00) | – |
|  | MMF-related AE | | 5 | (5.38) | 2 | (40.00) | 2.667 (0.361–19.712) |
|  | Other | | 15 | (16.13) | 5 | (33.33) | 2.000 (0.494–8.089) |
| eGFR (Japanese equation; mL/min/1.73 m^2^)^b^ | <30 | | 20 | (21.51) | 8 | (40.00) | Reference |
|  | ≥30 and ≤60 | | 63 | (67.74) | 14 | (22.22) | 0.429 (0.146–1.254) |
|  | >60 | | 10 | (10.75) | 1 | (10.00) | 0.167 (0.018–1.584) |
| eGFR (MDRD; mL/min/1.73 m^2^)^b^ | <30 | | 8 | (8.60) | 5 | (62.50) | Reference |
|  | ≥30 and ≤60 | | 45 | (48.39) | 13 | (28.89) | 0.244 (0.051–1.172) |
|  | >60 | | 40 | (43.01) | 5 | (12.50) | 0.086 (0.015–0.474) |
| eGFR (serum cystatin C; mL/min/1.73 m^2^)^b^ | <30 | | 14 | (15.05) | 5 | (35.71) | Reference |
|  | ≥30 and ≤60 | | 60 | (64.52) | 16 | (26.67) | 0.655 (0.191–2.248) |
|  | >60 | | 19 | (20.43) | 2 | (10.53) | 0.212 (0.034–1.318) |
| UPCR (g/gCr)^b^ | <0.55 | | 66 | (70.97) | 15 | (22.73) | Reference |
|  | ≥0.55 | | 6 | (6.45) | 0 | (0.00) | – |
|  | Unknown^a^ |  | 21 | (22.58) | 8 | (38.10) |  |
| Specific concomitant medication^c^ | No |  | 15 | (16.13) | 4 | (26.67) | Reference |
|  | Yes |  | 78 | (83.87) | 19 | (24.36) | 0.886 (0.252–3.109) |
| Concomitant antimetabolites | No |  | 9 | (9.68) | 2 | (22.22) | Reference |
|  | Yes |  | 84 | (90.32) | 21 | (25.00) | 1.167 (0.225–6.058) |
| Concomitant immunosuppressants | No |  | 0 | (0.00) | – |  | Reference |
|  | Yes |  | 93 | (100.00) | 23 | (24.73) | – |
| CNI dose reduction^d^ | No |  | 52 | (55.91) | 13 | (25.00) | Reference |
|  | Yes |  | 41 | (44.09) | 10 | (24.39) | 0.968 (0.374–2.502) |
| ^a^Shaded categories were not considered for tests; ^b^At the start of EVR treatment; ^c^Angiotensin II receptor antagonists, angiotensin-converting enzyme inhibitors, treatment drugs for dyslipidemia, and treatment drugs for diabetes mellitus including insulin; ^d^Patients with CNI dose reduction were defined as those in whom the dosage of CNIs was reduced by ≥30% relative to the dose at the start of treatment at ≥2 time points out of all assessment points.  AE, adverse event; CI, confidence interval; CNI, calcineurin inhibitor; eGFR, estimated glomerular filtration rate; EVR, everolimus; HLA, human leukocyte antigen; MDRD, modification of diet in renal disease; MMF, mycophenolate mofetil; OR, odds ratio; Tx, transplantation; UPCR, urinary protein/creatinine ratio | | | | | | | |
